# The results of Wisconsin Card Sorting Test in patients under forensic observation of their mental states in violent and non-violent subgroups

**DOI:** 10.1192/j.eurpsy.2024.1212

**Published:** 2024-08-27

**Authors:** N. Kőszegi, A. Lisincki, B. Baran, É. Jekkel

**Affiliations:** ^1^Department of Paediatrics; ^2^Department of Psychiatry and Psychotherapy, Semmelweis University, Budapest, Hungary

## Abstract

**Introduction:**

Previous studies showed, that reduced executive function can be associated with antisocial and aggressive behavior. For the measurement of executive functions numerous standardized neuropsychological tests are available.

**Objectives:**

We thought to compare the results of an executive function examination with Wisconsin Card Sorting Test (WCST) of patients observed at the Semmelweis University’s Department of Psychiatry and Psychotherapy to normative data from published database. We also performed a subgroup analysis between the violent and non-violent groups of the patients.

**Methods:**

After data clearance our dataset consisted of 20 patients, who were divided into two groups based on whether the crime they committed before their admission was violent according to the Cornell scale.The analyzed parameters were the number of perseverative errors, the percentage of perseverative errors, and the number of completed categories. For comparison, the data bank from the 1993 edition of the WCST manual as normative data was used. The deviation from the healthy average for all three parameters was compared between the violent and non-violent groups using a two-sample T-test.

**Results:**

There was significant difference between the patient and normal populations in all the 3 analyzed WCST parameters: the mean difference was 9,37+2,764, (p=0,0008) in the number of perseverative errors, 14,04+2,21 (p<0,0001) in the percentage of perseverative errors and -2,39+0,34 (p<0,0001) in the number of completed categories (Table 1).

**Table 1:** The difference between the average scores of healthy individuals grouped by age (from the 1993 WCST manual) and the scores of the patients.

Observed parameter	Average difference (Patient-normal)	SD	Confidence interval (95%)	P value
number of completed categories	-2,39	0,343	-3,064 ─ 1,716	<0,0001
number of perseverative errors	9,37	2,764	3,936 ─ 14,804	0,0008
percentage of perseverative errors	14,04	2,212	9,692─18,388	<0,0001

On the other hand, there were no significant differences between the violent and non-violent subgroups in the average deviations (from the normative data) of the number of perseverative errors, the percentage of perseverative errors and the number of completed categories (with p-values of 0.092, 0.34 and 0.59, respectively).

**Conclusions:**

As a limitation, it is important to note that due to the low sample size, and our sample’s heterogeneity in terms of psychiatric diagnosis, drawing reliable conclusions is not possible. However, our results were in line with previous similar research in the forensic psychiatric population (though not under forensic mental state observation) regarding the significant deviations in two examined WCST parameters when compared to normative data. Additionally our study did not find significant difference between the violent and non-violent subgroups of the patients.

**Disclosure of Interest:**

None Declared

